# 3-(5-Phenyl-4-phenyl­sulfonyl-1-*p*-tolyl-1*H*-pyrazol-3-yl)-1,2-dihydro­quinoxaline

**DOI:** 10.1107/S1600536811005940

**Published:** 2011-02-23

**Authors:** Hatem A. Abdel-Aziz, Ahmed Bari, Seik Weng Ng

**Affiliations:** a Department of Pharmaceutical Chemistry, College of Pharmacy, King Saud University, Riyadh 11451, Saudi Arabia; b Department of Chemistry, University of Malaya, 50603 Kuala Lumpur, Malaysia

## Abstract

In the crystal structure of the title compound, C_30_H_24_N_4_O_2_S, the dihydro­quinoxaline fused-ring system is disordered over three orientations in a 0.358 (2):0.318 (3):0.324 (3) ratio; the mean planes of the non-H atoms of the disorder components are aligned at 4.0 (3), 11.8 (4) and 41.7 (2)° with respect to the pyrazole ring. The rings of the phenyl and tolyl substituents are aligned at 64.0 (1) and 43.7 (1)° with respect to the pyrazole ring. Weak intermolecular C—H⋯O hydrogen bonding links the mol­ecules, forming supra­molecular chains running along the *a* axis.

## Related literature

For background to the biological properties of aryl-substituted pyrazoles, see: Abdel-Aziz *et al.* (2010 ▶).
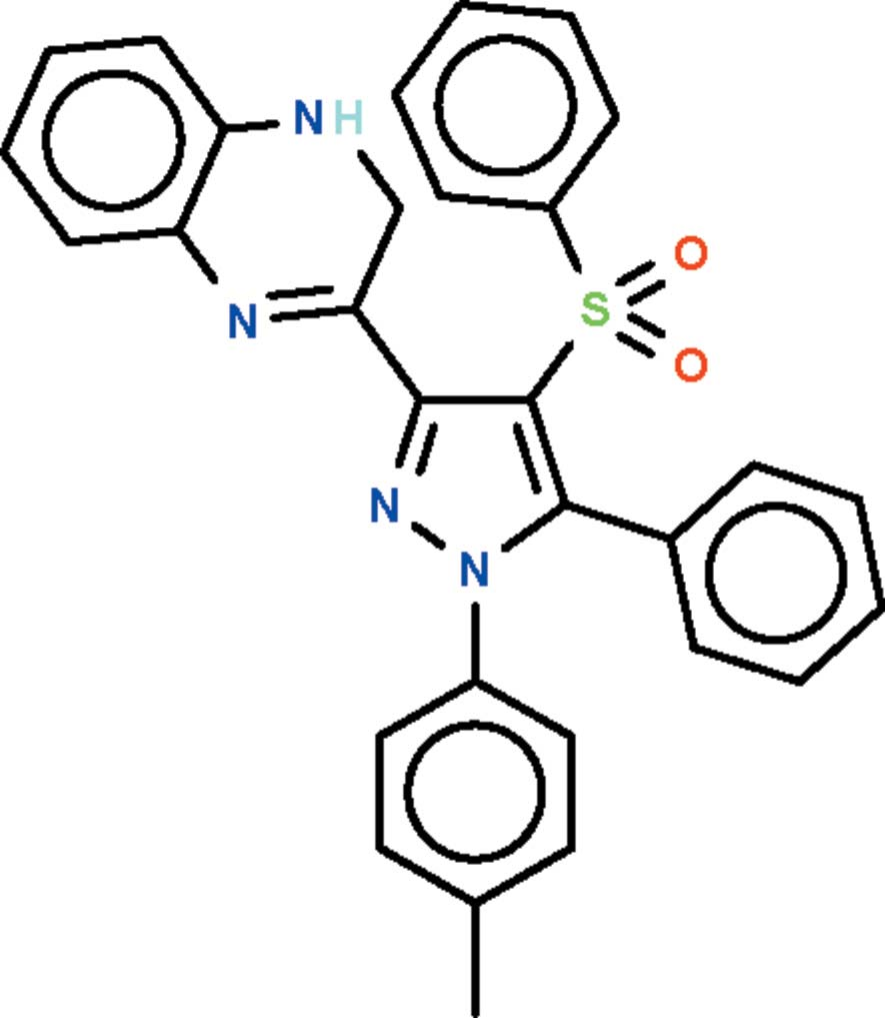


## Experimental

### 

#### Crystal data

C_30_H_24_N_4_O_2_S
*M* *_r_* = 504.59
Monoclinic, 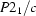

*a* = 6.2829 (2) Å
*b* = 24.5162 (7) Å
*c* = 15.9180 (5) Å
β = 91.925 (3)°
*V* = 2450.51 (13) Å^3^
*Z* = 4
Mo *K*α radiation
μ = 0.17 mm^−1^
*T* = 100 K
0.20 × 0.02 × 0.02 mm

#### Data collection

Agilent SuperNova Dual diffractometer with an Atlas detector
Absorption correction: multi-scan (*CrysAlis PRO*; Agilent, 2010 ▶) *T*_min_ = 0.967, *T*_max_ = 0.997
16288 measured reflections
5524 independent reflections
3897 reflections with *I* > 2σ(*I*)
*R*_int_ = 0.054

#### Refinement

*R*[*F* ^2^ > 2σ(*F* ^2^)] = 0.062
*wR*(*F* ^2^) = 0.158
*S* = 1.02
5524 reflections
422 parameters
139 restraints
H-atom parameters constrained
Δρ_max_ = 0.38 e Å^−3^
Δρ_min_ = −0.40 e Å^−3^

### 

Data collection: *CrysAlis PRO* (Agilent, 2010 ▶); cell refinement: *CrysAlis PRO*; data reduction: *CrysAlis PRO*; program(s) used to solve structure: *SHELXS97* (Sheldrick, 2008 ▶); program(s) used to refine structure: *SHELXL97* (Sheldrick, 2008 ▶); molecular graphics: *X-SEED* (Barbour, 2001 ▶); software used to prepare material for publication: *publCIF* (Westrip, 2010 ▶).

## Supplementary Material

Crystal structure: contains datablocks global, I. DOI: 10.1107/S1600536811005940/xu5163sup1.cif

Structure factors: contains datablocks I. DOI: 10.1107/S1600536811005940/xu5163Isup2.hkl

Additional supplementary materials:  crystallographic information; 3D view; checkCIF report

## Figures and Tables

**Table 1:** Hydrogen-bond geometry (Å, °)

*D*—H⋯*A*	*D*—H	H⋯*A*	*D*⋯*A*	*D*—H⋯*A*
C19—H19⋯O1^i^	0.95	2.57	3.430 (3)	150

Symmetry code: (i) 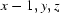.

**Fig. 1.**
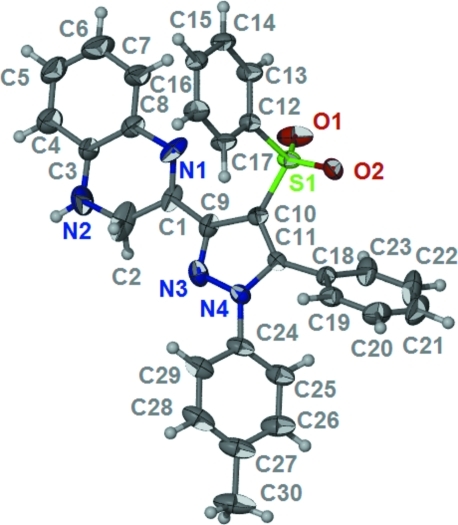
Thermal ellipsoid plot (Barbour, 2001) of C30H24N4O2S at the 70% probability level; hydrogen atoms are drawn as spheres of arbitrary radius. The disorder is not shown.

## supplementary crystallographic information

### Comment

We have reported the antitumor activity of aryl-pyrazoles against CaCo-2 and HEP-2 cell lines (Abdel-Aziz *et al.*, 2010). These compounds were synthesized by a cycloaddition under microwave conditions. The study is now extended to the synthesis of a pyrazole having a dihydroquinoxaline substituent (Scheme I). The dihydroquinoxalinyl subsitutent of the pyrazolyl ring of C_30_H_24_N_4_O_2_S adopts three orientations. The orientations refined to a 0.358 (2): 0.318 (3): 0.324 (3) ratio. The mean planes of the atoms passing through the non-hydrogen atoms of the disorder components are aligned at 4.0 (3), 11.8 (4) and 41.7 (2)° with respect to the five-membered ring (Fig. 1).

### Experimental

The acetyl portion of 1-[5-phenyl-4-(phenylfulonyl)-1-*p*-tolyl-1*H*-pyrazol-3-yl]ethane was first converted to a bromoacetyl unit by bromination at 363–373 K to yield 2-bromo-1-[5-phenyl-4-(phenylsulfonyl)-1-*p*-tolyl-1*H*-pyrazol-3-yl)ethanone. The compound (10 mmol) was heated with *o*-phenylenediamine (10 mmol) in ethanol to yield the title compound. Crystals were obtained upon recrystallization from ethanol.

### Refinement

Carbon-bound H-atoms were placed in calculated positions [C—H 0.95 to 0.98 Å, *U*_iso_(H) 1.2 to 1.5*U*_eq_(C)] and were included in the refinement in the riding model approximation.

The dihydroquinoxalinyl fused-ring is disordered over three orientations in a 0.358 (2): 0.318 (3): 0.324 (3) ratio. The first two disorder components are close to each other, so that the temperature factors of the singly-primed atoms were set to those of the unprimed atoms. The anisotropic temperature factors of the atoms of the three disorder components were restrained to be nearly isotropic, with the restraint being much tighter for the C1/C1'/C1" set of atoms. The aromatic rings were refined as rigid hexagons of 1.39 Å sides. The carbon–nitrogen_tertiary_ distances were restrained to 1.35±0.01 Å and the carbon–nitrogen_secondary_ to 1.45±0.01 Å. The carbon–carbon single-bond distances were restrained to 1.50±0.01 Å. The amino H-atoms were treated in the riding mode [N—H 0.88 Å, *U*_iso_(H) 1.2*U*_eq_(N)].

### Crystal data

**Table:**

C_30_H_24_N_4_O_2_S	*F*(000) = 1056
*M**_r_* = 504.59	*D*_x_ = 1.368 Mg m^−^^3^
Monoclinic, *P*2_1_/*c*	Mo *K*α radiation, λ = 0.71073 Å
Hall symbol: -P 2ybc	Cell parameters from 4169 reflections
*a* = 6.2829 (2) Å	θ = 2.5–29.3°
*b* = 24.5162 (7) Å	µ = 0.17 mm^−^^1^
*c* = 15.9180 (5) Å	*T* = 100 K
β = 91.925 (3)°	Prism, yellow
*V* = 2450.51 (13) Å^3^	0.20 × 0.02 × 0.02 mm
*Z* = 4	

### Data collection

**Table:**

Agilent SuperNova Dual diffractometer with an Atlas detector	5524 independent reflections
Radiation source: SuperNova (Mo) X-ray Source	3897 reflections with *I* > 2σ(*I*)
Mirror	*R*_int_ = 0.054
Detector resolution: 10.4041 pixels mm^-1^	θ_max_ = 27.5°, θ_min_ = 2.6°
ω scans	*h* = −8→8
Absorption correction: multi-scan (*CrysAlis PRO*; Agilent, 2010)	*k* = −31→31
*T*_min_ = 0.967, *T*_max_ = 0.997	*l* = −20→20
16288 measured reflections	

### Refinement

**Table:**

Refinement on *F*^2^	Primary atom site location: structure-invariant direct methods
Least-squares matrix: full	Secondary atom site location: difference Fourier map
*R*[*F*^2^ > 2σ(*F*^2^)] = 0.062	Hydrogen site location: inferred from neighbouring sites
*wR*(*F*^2^) = 0.158	H-atom parameters constrained
*S* = 1.02	*w* = 1/[σ^2^(*F*_o_^2^) + (0.0634*P*)^2^ + 1.9372*P*] where *P* = (*F*_o_^2^ + 2*F*_c_^2^)/3
5524 reflections	(Δ/σ)_max_ = 0.001
422 parameters	Δρ_max_ = 0.38 e Å^−^^3^
139 restraints	Δρ_min_ = −0.40 e Å^−^^3^

### Fractional atomic coordinates and isotropic or equivalent isotropic displacement parameters (Å^2^)

**Table:**

	*x*	*y*	*z*	*U*_iso_*/*U*_eq_	Occ. (<1)
S1	0.81495 (10)	0.59167 (2)	0.63024 (4)	0.02549 (18)	
O1	1.0308 (3)	0.59391 (8)	0.66188 (15)	0.0434 (6)	
O2	0.7677 (3)	0.62026 (7)	0.55309 (12)	0.0317 (5)	
N1	0.804 (2)	0.5182 (4)	0.8067 (8)	0.034 (3)	0.358 (2)
N2	0.6304 (13)	0.5044 (4)	0.9751 (5)	0.0518 (19)	0.358 (2)
H2	0.5832	0.5024	1.0264	0.062*	0.358 (2)
N1'	0.854 (3)	0.5300 (7)	0.8110 (12)	0.034 (3)	0.318 (3)
N2'	0.7437 (15)	0.5043 (5)	0.9645 (7)	0.0518 (19)	0.318 (3)
H2'	0.6985	0.4916	1.0125	0.062*	0.318 (3)
N1"	0.8972 (12)	0.5346 (4)	0.7959 (8)	0.018 (2)	0.324 (3)
N2"	0.6519 (13)	0.4503 (3)	0.8722 (6)	0.058 (3)	0.324 (3)
H2"	0.5796	0.4244	0.8967	0.070*	0.324 (3)
N3	0.4322 (4)	0.62374 (9)	0.81706 (14)	0.0300 (5)	
N4	0.3835 (3)	0.66333 (8)	0.76021 (13)	0.0224 (5)	
C1	0.696 (2)	0.5572 (4)	0.8435 (7)	0.027 (2)	0.358 (2)
C2	0.5687 (19)	0.5421 (6)	0.9157 (8)	0.049 (3)	0.358 (2)
H2A	0.5418	0.5764	0.9465	0.059*	0.358 (2)
H2B	0.4290	0.5298	0.8922	0.059*	0.358 (2)
C3	0.7815 (8)	0.4692 (2)	0.9407 (3)	0.0328 (14)	0.358 (2)
C4	0.8492 (9)	0.4250 (2)	0.9894 (3)	0.0443 (16)	0.358 (2)
H4A	0.7923	0.4194	1.0433	0.053*	0.358 (2)
C5	1.0001 (10)	0.3892 (2)	0.9594 (3)	0.0458 (17)	0.358 (2)
H5	1.0464	0.3590	0.9927	0.055*	0.358 (2)
C6	1.0834 (10)	0.3974 (2)	0.8806 (4)	0.054 (2)	0.358 (2)
H6	1.1865	0.3729	0.8600	0.065*	0.358 (2)
C7	1.0157 (10)	0.4416 (2)	0.8318 (3)	0.0432 (18)	0.358 (2)
H7	1.0726	0.4472	0.7780	0.052*	0.358 (2)
C8	0.8647 (9)	0.47745 (19)	0.8619 (3)	0.0314 (16)	0.358 (2)
C1'	0.659 (2)	0.5469 (5)	0.8348 (9)	0.027 (2)	0.318 (3)
C2'	0.634 (2)	0.5474 (6)	0.9271 (9)	0.049 (3)	0.318 (3)
H2'A	0.6885	0.5822	0.9506	0.059*	0.318 (3)
H2'B	0.4809	0.5446	0.9396	0.059*	0.318 (3)
C3'	0.9213 (9)	0.4799 (2)	0.9307 (4)	0.0328 (14)	0.318 (3)
C4'	1.0383 (11)	0.4432 (3)	0.9802 (3)	0.0443 (16)	0.318 (3)
H4'	0.9969	0.4356	1.0358	0.053*	0.318 (3)
C5'	1.2157 (10)	0.4176 (3)	0.9483 (4)	0.0458 (17)	0.318 (3)
H5'	1.2957	0.3925	0.9821	0.055*	0.318 (3)
C6'	1.2763 (11)	0.4288 (3)	0.8669 (4)	0.054 (2)	0.318 (3)
H6'	1.3976	0.4113	0.8451	0.065*	0.318 (3)
C7'	1.1594 (14)	0.4655 (4)	0.8174 (3)	0.0432 (18)	0.318 (3)
H7'	1.2007	0.4731	0.7617	0.052*	0.318 (3)
C8'	0.9819 (12)	0.4911 (3)	0.8493 (4)	0.0314 (16)	0.318 (3)
C1"	0.6918 (14)	0.5421 (4)	0.8112 (7)	0.030 (3)	0.324 (3)
C2"	0.5589 (15)	0.4998 (3)	0.8485 (6)	0.042 (2)	0.324 (3)
H2"A	0.4948	0.5157	0.8989	0.050*	0.324 (3)
H2"B	0.4406	0.4917	0.8078	0.050*	0.324 (3)
C3"	0.8676 (10)	0.4445 (3)	0.8547 (5)	0.053 (3)	0.324 (3)
C4"	0.9628 (12)	0.3945 (3)	0.8730 (6)	0.065 (4)	0.324 (3)
H4"	0.8816	0.3659	0.8962	0.078*	0.324 (3)
C5"	1.1768 (13)	0.3865 (3)	0.8573 (6)	0.075 (4)	0.324 (3)
H5"	1.2419	0.3523	0.8698	0.089*	0.324 (3)
C6"	1.2956 (10)	0.4284 (3)	0.8234 (6)	0.065 (4)	0.324 (3)
H6"	1.4419	0.4229	0.8126	0.078*	0.324 (3)
C7"	1.2003 (12)	0.4783 (3)	0.8051 (5)	0.051 (3)	0.324 (3)
H7"	1.2815	0.5070	0.7819	0.061*	0.324 (3)
C8"	0.9863 (12)	0.4864 (2)	0.8208 (5)	0.044 (3)	0.324 (3)
C9	0.5873 (4)	0.59504 (10)	0.78359 (17)	0.0275 (6)	
C10	0.6366 (4)	0.61620 (9)	0.70379 (16)	0.0213 (5)	
C11	0.5021 (4)	0.66014 (9)	0.69050 (15)	0.0193 (5)	
C12	0.7410 (4)	0.52265 (10)	0.61533 (16)	0.0222 (5)	
C13	0.9002 (4)	0.48606 (10)	0.59728 (18)	0.0306 (6)	
H13	1.0449	0.4974	0.5986	0.037*	
C14	0.8469 (4)	0.43271 (11)	0.57721 (18)	0.0313 (6)	
H14	0.9554	0.4075	0.5637	0.038*	
C15	0.6385 (4)	0.41603 (10)	0.57666 (17)	0.0279 (6)	
H15	0.6029	0.3793	0.5633	0.034*	
C16	0.4805 (4)	0.45288 (10)	0.5956 (2)	0.0336 (7)	
H16	0.3364	0.4412	0.5960	0.040*	
C17	0.5304 (4)	0.50657 (10)	0.61392 (18)	0.0299 (6)	
H17	0.4213	0.5321	0.6254	0.036*	
C18	0.4762 (4)	0.69961 (9)	0.62105 (16)	0.0230 (5)	
C19	0.2844 (4)	0.70325 (11)	0.57537 (16)	0.0279 (6)	
H19	0.1721	0.6785	0.5860	0.033*	
C20	0.2575 (5)	0.74308 (13)	0.51430 (19)	0.0420 (8)	
H20	0.1263	0.7456	0.4831	0.050*	
C21	0.4199 (7)	0.77902 (13)	0.4985 (2)	0.0522 (9)	
H21	0.3998	0.8068	0.4575	0.063*	
C22	0.6139 (6)	0.77443 (13)	0.5431 (2)	0.0519 (9)	
H22	0.7273	0.7986	0.5312	0.062*	
C23	0.6420 (5)	0.73508 (11)	0.60416 (18)	0.0352 (7)	
H23	0.7741	0.7322	0.6347	0.042*	
C24	0.2196 (4)	0.70160 (11)	0.77998 (16)	0.0273 (6)	
C25	0.2472 (4)	0.75683 (11)	0.76539 (19)	0.0338 (6)	
H25	0.3723	0.7698	0.7401	0.041*	
C26	0.0889 (5)	0.79293 (13)	0.7884 (2)	0.0409 (7)	
H26	0.1070	0.8309	0.7790	0.049*	
C27	−0.0956 (4)	0.77443 (13)	0.8251 (2)	0.0406 (8)	
C28	−0.1194 (4)	0.71885 (13)	0.83758 (19)	0.0383 (7)	
H28	−0.2454	0.7057	0.8620	0.046*	
C29	0.0362 (4)	0.68207 (12)	0.81537 (17)	0.0310 (6)	
H29	0.0174	0.6441	0.8242	0.037*	
C30	−0.2606 (5)	0.81477 (15)	0.8538 (2)	0.0539 (10)	
H30A	−0.3979	0.7962	0.8585	0.081*	
H30B	−0.2750	0.8445	0.8129	0.081*	
H30C	−0.2157	0.8297	0.9087	0.081*	

### Atomic displacement parameters (Å^2^)

**Table:**

	*U*^11^	*U*^22^	*U*^33^	*U*^12^	*U*^13^	*U*^23^
S1	0.0197 (3)	0.0221 (3)	0.0349 (4)	−0.0019 (2)	0.0043 (3)	−0.0087 (3)
O1	0.0207 (9)	0.0356 (11)	0.0736 (16)	−0.0014 (8)	−0.0025 (10)	−0.0245 (11)
O2	0.0408 (11)	0.0249 (9)	0.0305 (10)	−0.0031 (8)	0.0153 (9)	−0.0025 (8)
N1	0.061 (6)	0.015 (5)	0.025 (3)	0.003 (4)	−0.019 (4)	−0.008 (3)
N2	0.062 (5)	0.062 (3)	0.033 (3)	0.014 (5)	0.013 (4)	0.021 (3)
N1'	0.061 (6)	0.015 (5)	0.025 (3)	0.003 (4)	−0.019 (4)	−0.008 (3)
N2'	0.062 (5)	0.062 (3)	0.033 (3)	0.014 (5)	0.013 (4)	0.021 (3)
N1"	0.017 (3)	0.021 (4)	0.018 (4)	−0.001 (3)	0.011 (3)	0.007 (3)
N2"	0.081 (6)	0.034 (4)	0.062 (6)	0.000 (4)	0.027 (5)	0.017 (4)
N3	0.0298 (12)	0.0392 (13)	0.0211 (11)	−0.0052 (10)	0.0004 (9)	0.0040 (10)
N4	0.0209 (10)	0.0268 (10)	0.0193 (10)	−0.0008 (8)	0.0009 (8)	−0.0030 (9)
C1	0.044 (4)	0.017 (4)	0.021 (3)	−0.011 (3)	−0.014 (3)	−0.003 (3)
C2	0.068 (7)	0.044 (4)	0.033 (4)	−0.022 (4)	−0.033 (4)	0.010 (3)
C3	0.041 (3)	0.031 (3)	0.026 (3)	0.008 (3)	0.004 (3)	0.007 (2)
C4	0.052 (4)	0.041 (3)	0.040 (3)	0.010 (3)	0.003 (3)	0.007 (3)
C5	0.054 (4)	0.037 (3)	0.045 (4)	0.013 (3)	−0.006 (3)	0.013 (3)
C6	0.055 (5)	0.048 (4)	0.058 (5)	0.028 (4)	−0.005 (4)	0.006 (4)
C7	0.049 (4)	0.042 (4)	0.039 (4)	0.022 (3)	0.006 (4)	0.009 (3)
C8	0.031 (4)	0.026 (3)	0.038 (4)	0.002 (3)	−0.002 (3)	0.002 (3)
C1'	0.044 (4)	0.017 (4)	0.021 (3)	−0.011 (3)	−0.014 (3)	−0.003 (3)
C2'	0.068 (7)	0.044 (4)	0.033 (4)	−0.022 (4)	−0.033 (4)	0.010 (3)
C3'	0.041 (3)	0.031 (3)	0.026 (3)	0.008 (3)	0.004 (3)	0.007 (2)
C4'	0.052 (4)	0.041 (3)	0.040 (3)	0.010 (3)	0.003 (3)	0.007 (3)
C5'	0.054 (4)	0.037 (3)	0.045 (4)	0.013 (3)	−0.006 (3)	0.013 (3)
C6'	0.055 (5)	0.048 (4)	0.058 (5)	0.028 (4)	−0.005 (4)	0.006 (4)
C7'	0.049 (4)	0.042 (4)	0.039 (4)	0.022 (3)	0.006 (4)	0.009 (3)
C8'	0.031 (4)	0.026 (3)	0.038 (4)	0.002 (3)	−0.002 (3)	0.002 (3)
C1"	0.040 (4)	0.033 (5)	0.017 (4)	0.002 (4)	0.006 (4)	0.009 (4)
C2"	0.055 (5)	0.029 (4)	0.042 (5)	0.003 (4)	0.012 (4)	0.000 (4)
C3"	0.064 (6)	0.056 (6)	0.041 (5)	0.014 (5)	0.018 (5)	0.011 (5)
C4"	0.087 (8)	0.053 (6)	0.057 (6)	0.026 (6)	0.008 (6)	0.014 (5)
C5"	0.088 (8)	0.062 (7)	0.076 (7)	0.030 (6)	0.022 (6)	0.012 (6)
C6"	0.071 (7)	0.067 (7)	0.056 (7)	0.024 (6)	−0.001 (6)	0.012 (6)
C7"	0.049 (6)	0.050 (6)	0.054 (6)	0.023 (5)	0.001 (5)	0.000 (5)
C8"	0.058 (6)	0.042 (6)	0.033 (5)	0.017 (5)	0.003 (5)	0.009 (5)
C9	0.0296 (13)	0.0253 (13)	0.0274 (14)	−0.0026 (11)	−0.0031 (11)	0.0049 (11)
C10	0.0210 (12)	0.0188 (11)	0.0240 (13)	−0.0009 (9)	−0.0013 (10)	−0.0032 (10)
C11	0.0178 (11)	0.0213 (11)	0.0186 (12)	−0.0043 (9)	0.0001 (9)	−0.0045 (10)
C12	0.0223 (12)	0.0224 (12)	0.0220 (13)	0.0006 (10)	−0.0002 (10)	−0.0041 (10)
C13	0.0193 (12)	0.0294 (13)	0.0431 (17)	0.0014 (10)	0.0017 (12)	−0.0059 (12)
C14	0.0283 (14)	0.0254 (13)	0.0406 (16)	0.0070 (11)	0.0065 (12)	−0.0037 (12)
C15	0.0324 (14)	0.0199 (12)	0.0317 (15)	−0.0008 (10)	0.0049 (12)	−0.0048 (11)
C16	0.0223 (13)	0.0262 (13)	0.0528 (19)	−0.0042 (11)	0.0066 (13)	−0.0061 (13)
C17	0.0197 (12)	0.0225 (12)	0.0477 (17)	0.0029 (10)	0.0034 (12)	−0.0058 (12)
C18	0.0301 (13)	0.0190 (11)	0.0200 (13)	0.0008 (10)	0.0009 (10)	−0.0017 (10)
C19	0.0293 (13)	0.0302 (13)	0.0241 (14)	0.0045 (11)	−0.0007 (11)	−0.0040 (11)
C20	0.0542 (19)	0.0457 (18)	0.0258 (15)	0.0225 (16)	−0.0046 (14)	0.0014 (14)
C21	0.094 (3)	0.0345 (17)	0.0286 (17)	0.0108 (18)	0.0036 (18)	0.0107 (14)
C22	0.083 (3)	0.0366 (17)	0.0364 (18)	−0.0200 (17)	0.0049 (18)	0.0102 (15)
C23	0.0440 (17)	0.0307 (14)	0.0307 (15)	−0.0110 (13)	−0.0007 (13)	0.0031 (12)
C24	0.0213 (12)	0.0349 (14)	0.0257 (14)	0.0010 (11)	−0.0010 (10)	−0.0155 (12)
C25	0.0258 (13)	0.0356 (15)	0.0403 (16)	−0.0025 (12)	0.0038 (12)	−0.0164 (13)
C26	0.0339 (15)	0.0380 (16)	0.0503 (19)	0.0039 (13)	−0.0052 (14)	−0.0230 (15)
C27	0.0241 (14)	0.0551 (19)	0.0420 (18)	0.0051 (13)	−0.0048 (12)	−0.0297 (15)
C28	0.0223 (13)	0.060 (2)	0.0330 (16)	−0.0050 (13)	0.0014 (12)	−0.0234 (15)
C29	0.0223 (13)	0.0443 (16)	0.0261 (14)	−0.0013 (12)	−0.0025 (11)	−0.0129 (13)
C30	0.0335 (16)	0.064 (2)	0.064 (2)	0.0130 (15)	−0.0042 (16)	−0.0388 (19)

### Geometric parameters (Å, °)

**Table:**

S1—O1	1.432 (2)	C1"—C9	1.512 (8)
S1—O2	1.436 (2)	C2"—H2"A	0.9900
S1—C10	1.754 (2)	C2"—H2"B	0.9900
S1—C12	1.769 (2)	C3"—C4"	1.3900
N1—C1	1.322 (9)	C3"—C8"	1.3900
N1—C8	1.375 (9)	C4"—C5"	1.3900
N2—C2	1.370 (9)	C4"—H4"	0.9500
N2—C3	1.408 (7)	C5"—C6"	1.3900
N2—N2^i^	1.856 (16)	C5"—H5"	0.9500
N2—H2	0.8800	C6"—C7"	1.3900
N1'—C1'	1.358 (10)	C6"—H6"	0.9500
N1'—C8'	1.379 (9)	C7"—C8"	1.3900
N2'—C2'	1.385 (9)	C7"—H7"	0.9500
N2'—C3'	1.390 (8)	C9—C10	1.416 (4)
N2'—H2'	0.8800	C10—C11	1.381 (3)
N1"—C1"	1.335 (8)	C11—C18	1.474 (3)
N1"—C8"	1.361 (8)	C12—C13	1.381 (3)
N2"—C2"	1.392 (8)	C12—C17	1.380 (3)
N2"—C3"	1.400 (8)	C13—C14	1.385 (4)
N2"—H2"	0.8800	C13—H13	0.9500
N3—C9	1.328 (3)	C14—C15	1.371 (4)
N3—N4	1.355 (3)	C14—H14	0.9500
N4—C11	1.359 (3)	C15—C16	1.383 (4)
N4—C24	1.436 (3)	C15—H15	0.9500
C1—C2	1.469 (9)	C16—C17	1.382 (4)
C1—C9	1.480 (8)	C16—H16	0.9500
C2—H2A	0.9900	C17—H17	0.9500
C2—H2B	0.9900	C18—C19	1.389 (4)
C3—C4	1.3900	C18—C23	1.390 (4)
C3—C8	1.3900	C19—C20	1.384 (4)
C4—C5	1.3900	C19—H19	0.9500
C4—H4A	0.9500	C20—C21	1.378 (5)
C5—C6	1.3900	C20—H20	0.9500
C5—H5	0.9500	C21—C22	1.394 (5)
C6—C7	1.3900	C21—H21	0.9500
C6—H6	0.9500	C22—C23	1.377 (4)
C7—C8	1.3900	C22—H22	0.9500
C7—H7	0.9500	C23—H23	0.9500
C1'—C2'	1.483 (10)	C24—C29	1.385 (4)
C1'—C9	1.494 (9)	C24—C25	1.386 (4)
C2'—H2'A	0.9900	C25—C26	1.390 (4)
C2'—H2'B	0.9900	C25—H25	0.9500
C3'—C4'	1.3900	C26—C27	1.391 (4)
C3'—C8'	1.3900	C26—H26	0.9500
C4'—C5'	1.3900	C27—C28	1.386 (5)
C4'—H4'	0.9500	C27—C30	1.515 (4)
C5'—C6'	1.3900	C28—C29	1.384 (4)
C5'—H5'	0.9500	C28—H28	0.9500
C6'—C7'	1.3900	C29—H29	0.9500
C6'—H6'	0.9500	C30—H30A	0.9800
C7'—C8'	1.3900	C30—H30B	0.9800
C7'—H7'	0.9500	C30—H30C	0.9800
C1"—C2"	1.470 (9)		
			
O1—S1—O2	116.63 (13)	C8"—C3"—N2"	122.5 (6)
O1—S1—C10	111.78 (12)	C5"—C4"—C3"	120.0
O2—S1—C10	106.57 (11)	C5"—C4"—H4"	120.0
O1—S1—C12	108.98 (12)	C3"—C4"—H4"	120.0
O2—S1—C12	107.84 (11)	C4"—C5"—C6"	120.0
C10—S1—C12	104.29 (12)	C4"—C5"—H5"	120.0
C1—N1—C8	112.2 (10)	C6"—C5"—H5"	120.0
C2—N2—C3	108.9 (9)	C7"—C6"—C5"	120.0
C2—N2—N2^i^	98.0 (8)	C7"—C6"—H6"	120.0
C3—N2—N2^i^	135.4 (9)	C5"—C6"—H6"	120.0
C2—N2—H2	125.5	C8"—C7"—C6"	120.0
C3—N2—H2	125.5	C8"—C7"—H7"	120.0
C1'—N1'—C8'	127.4 (13)	C6"—C7"—H7"	120.0
C2'—N2'—C3'	123.8 (10)	N1"—C8"—C7"	117.5 (6)
C2'—N2'—H2'	118.1	N1"—C8"—C3"	122.3 (6)
C3'—N2'—H2'	118.1	C7"—C8"—C3"	120.0
C1"—N1"—C8"	117.2 (7)	N3—C9—C10	110.6 (2)
C2"—N2"—C3"	115.8 (7)	N3—C9—C1	113.7 (6)
C2"—N2"—H2"	122.1	C10—C9—C1	134.2 (6)
C3"—N2"—H2"	122.1	N3—C9—C1'	114.3 (7)
C9—N3—N4	105.3 (2)	C10—C9—C1'	135.1 (7)
N3—N4—C11	112.7 (2)	N3—C9—C1"	130.9 (4)
N3—N4—C24	118.0 (2)	C10—C9—C1"	118.0 (4)
C11—N4—C24	129.3 (2)	C11—C10—C9	105.8 (2)
N1—C1—C2	117.8 (10)	C11—C10—S1	124.51 (19)
N1—C1—C9	113.7 (8)	C9—C10—S1	129.57 (19)
C2—C1—C9	114.3 (8)	N4—C11—C10	105.6 (2)
N2—C2—C1	124.2 (11)	N4—C11—C18	121.6 (2)
N2—C2—H2A	106.3	C10—C11—C18	132.7 (2)
C1—C2—H2A	106.3	C13—C12—C17	120.8 (2)
N2—C2—H2B	106.3	C13—C12—S1	117.43 (19)
C1—C2—H2B	106.3	C17—C12—S1	121.52 (19)
H2A—C2—H2B	106.4	C12—C13—C14	119.3 (2)
C4—C3—C8	120.0	C12—C13—H13	120.3
C4—C3—N2	117.2 (5)	C14—C13—H13	120.3
C8—C3—N2	122.8 (5)	C15—C14—C13	120.5 (2)
C5—C4—C3	120.0	C15—C14—H14	119.8
C5—C4—H4A	120.0	C13—C14—H14	119.8
C3—C4—H4A	120.0	C14—C15—C16	119.7 (2)
C4—C5—C6	120.0	C14—C15—H15	120.1
C4—C5—H5	120.0	C16—C15—H15	120.1
C6—C5—H5	120.0	C15—C16—C17	120.6 (2)
C7—C6—C5	120.0	C15—C16—H16	119.7
C7—C6—H6	120.0	C17—C16—H16	119.7
C5—C6—H6	120.0	C12—C17—C16	119.1 (2)
C6—C7—C8	120.0	C12—C17—H17	120.5
C6—C7—H7	120.0	C16—C17—H17	120.5
C8—C7—H7	120.0	C19—C18—C23	120.1 (2)
N1—C8—C7	114.6 (7)	C19—C18—C11	120.5 (2)
N1—C8—C3	125.3 (7)	C23—C18—C11	119.4 (2)
C7—C8—C3	120.0	C20—C19—C18	119.8 (3)
N1'—C1'—C2'	113.9 (12)	C20—C19—H19	120.1
N1'—C1'—C9	110.2 (9)	C18—C19—H19	120.1
C2'—C1'—C9	119.7 (10)	C21—C20—C19	120.4 (3)
N2'—C2'—C1'	110.7 (11)	C21—C20—H20	119.8
N2'—C2'—H2'A	109.5	C19—C20—H20	119.8
C1'—C2'—H2'A	109.5	C20—C21—C22	119.8 (3)
N2'—C2'—H2'B	109.5	C20—C21—H21	120.1
C1'—C2'—H2'B	109.5	C22—C21—H21	120.1
H2'A—C2'—H2'B	108.1	C23—C22—C21	120.3 (3)
C4'—C3'—C8'	120.0	C23—C22—H22	119.8
C4'—C3'—N2'	118.3 (6)	C21—C22—H22	119.8
C8'—C3'—N2'	121.7 (6)	C22—C23—C18	119.7 (3)
C3'—C4'—C5'	120.0	C22—C23—H23	120.1
C3'—C4'—H4'	120.0	C18—C23—H23	120.1
C5'—C4'—H4'	120.0	C29—C24—C25	121.1 (2)
C4'—C5'—C6'	120.0	C29—C24—N4	118.4 (2)
C4'—C5'—H5'	120.0	C25—C24—N4	120.4 (2)
C6'—C5'—H5'	120.0	C24—C25—C26	118.9 (3)
C7'—C6'—C5'	120.0	C24—C25—H25	120.6
C7'—C6'—H6'	120.0	C26—C25—H25	120.6
C5'—C6'—H6'	120.0	C25—C26—C27	121.1 (3)
C6'—C7'—C8'	120.0	C25—C26—H26	119.4
C6'—C7'—H7'	120.0	C27—C26—H26	119.4
C8'—C7'—H7'	120.0	C28—C27—C26	118.4 (3)
N1'—C8'—C7'	128.0 (9)	C28—C27—C30	121.4 (3)
N1'—C8'—C3'	112.0 (9)	C26—C27—C30	120.1 (3)
C7'—C8'—C3'	120.0	C29—C28—C27	121.6 (3)
N1"—C1"—C2"	122.8 (7)	C29—C28—H28	119.2
N1"—C1"—C9	118.6 (7)	C27—C28—H28	119.2
C2"—C1"—C9	118.5 (7)	C24—C29—C28	118.9 (3)
N2"—C2"—C1"	119.1 (8)	C24—C29—H29	120.6
N2"—C2"—H2"A	107.5	C28—C29—H29	120.6
C1"—C2"—H2"A	107.5	C27—C30—H30A	109.5
N2"—C2"—H2"B	107.5	C27—C30—H30B	109.5
C1"—C2"—H2"B	107.5	H30A—C30—H30B	109.5
H2"A—C2"—H2"B	107.0	C27—C30—H30C	109.5
C4"—C3"—C8"	120.0	H30A—C30—H30C	109.5
C4"—C3"—N2"	117.5 (6)	H30B—C30—H30C	109.5
			
C9—N3—N4—C11	0.9 (3)	C2'—C1'—C9—N3	−26.6 (14)
C9—N3—N4—C24	−179.4 (2)	N1'—C1'—C9—C10	23.1 (17)
C8—N1—C1—C2	−29.4 (15)	C2'—C1'—C9—C10	158.1 (8)
C8—N1—C1—C9	−167.0 (9)	N1'—C1'—C9—C1	−71 (4)
C3—N2—C2—C1	−22.3 (15)	C2'—C1'—C9—C1	64 (4)
N2^i^—N2—C2—C1	−166.2 (11)	N1'—C1'—C9—C1"	32 (2)
N1—C1—C2—N2	37.2 (17)	C2'—C1'—C9—C1"	167 (4)
C9—C1—C2—N2	174.5 (10)	N1"—C1"—C9—N3	−147.6 (10)
C2—N2—C3—C4	−174.5 (7)	C2"—C1"—C9—N3	36.5 (13)
N2^i^—N2—C3—C4	−50.6 (14)	N1"—C1"—C9—C10	41.6 (14)
C2—N2—C3—C8	6.3 (11)	C2"—C1"—C9—C10	−134.3 (8)
N2^i^—N2—C3—C8	130.2 (11)	N1"—C1"—C9—C1	−96 (2)
C8—C3—C4—C5	0.0	C2"—C1"—C9—C1	87.9 (17)
N2—C3—C4—C5	−179.2 (7)	N1"—C1"—C9—C1'	−132 (3)
C3—C4—C5—C6	0.0	C2"—C1"—C9—C1'	53 (2)
C4—C5—C6—C7	0.0	N3—C9—C10—C11	0.3 (3)
C5—C6—C7—C8	0.0	C1—C9—C10—C11	−164.4 (7)
C1—N1—C8—C7	−167.1 (8)	C1'—C9—C10—C11	175.7 (9)
C1—N1—C8—C3	15.5 (14)	C1"—C9—C10—C11	172.9 (5)
C6—C7—C8—N1	−177.5 (8)	N3—C9—C10—S1	−175.66 (19)
C6—C7—C8—C3	0.0	C1—C9—C10—S1	19.6 (7)
C4—C3—C8—N1	177.3 (8)	C1'—C9—C10—S1	−0.3 (9)
N2—C3—C8—N1	−3.6 (10)	C1"—C9—C10—S1	−3.1 (6)
C4—C3—C8—C7	0.0	O1—S1—C10—C11	119.9 (2)
N2—C3—C8—C7	179.2 (7)	O2—S1—C10—C11	−8.6 (2)
C8'—N1'—C1'—C2'	34 (3)	C12—S1—C10—C11	−122.5 (2)
C8'—N1'—C1'—C9	172.2 (17)	O1—S1—C10—C9	−64.8 (3)
C3'—N2'—C2'—C1'	26.5 (18)	O2—S1—C10—C9	166.7 (2)
N1'—C1'—C2'—N2'	−35.5 (18)	C12—S1—C10—C9	52.8 (3)
C9—C1'—C2'—N2'	−169.0 (11)	N3—N4—C11—C10	−0.7 (3)
C2'—N2'—C3'—C4'	169.7 (10)	C24—N4—C11—C10	179.7 (2)
C2'—N2'—C3'—C8'	−10.8 (16)	N3—N4—C11—C18	−179.4 (2)
C8'—C3'—C4'—C5'	0.0	C24—N4—C11—C18	0.9 (4)
N2'—C3'—C4'—C5'	179.4 (8)	C9—C10—C11—N4	0.2 (3)
C3'—C4'—C5'—C6'	0.0	S1—C10—C11—N4	176.44 (17)
C4'—C5'—C6'—C7'	0.0	C9—C10—C11—C18	178.8 (2)
C5'—C6'—C7'—C8'	0.0	S1—C10—C11—C18	−5.0 (4)
C1'—N1'—C8'—C7'	165.9 (14)	O1—S1—C12—C13	−28.8 (3)
C1'—N1'—C8'—C3'	−17 (2)	O2—S1—C12—C13	98.7 (2)
C6'—C7'—C8'—N1'	176.9 (14)	C10—S1—C12—C13	−148.3 (2)
C6'—C7'—C8'—C3'	0.0	O1—S1—C12—C17	157.2 (2)
C4'—C3'—C8'—N1'	−177.4 (12)	O2—S1—C12—C17	−75.4 (2)
N2'—C3'—C8'—N1'	3.2 (13)	C10—S1—C12—C17	37.7 (3)
C4'—C3'—C8'—C7'	0.0	C17—C12—C13—C14	0.3 (4)
N2'—C3'—C8'—C7'	−179.4 (9)	S1—C12—C13—C14	−173.9 (2)
C8"—N1"—C1"—C2"	−4(2)	C12—C13—C14—C15	−1.1 (4)
C8"—N1"—C1"—C9	−179.4 (10)	C13—C14—C15—C16	0.5 (4)
C3"—N2"—C2"—C1"	−2.6 (15)	C14—C15—C16—C17	0.9 (4)
N1"—C1"—C2"—N2"	2.7 (18)	C13—C12—C17—C16	1.2 (4)
C9—C1"—C2"—N2"	178.4 (9)	S1—C12—C17—C16	175.0 (2)
C2"—N2"—C3"—C4"	−176.3 (7)	C15—C16—C17—C12	−1.8 (5)
C2"—N2"—C3"—C8"	3.9 (12)	N4—C11—C18—C19	−63.1 (3)
C8"—C3"—C4"—C5"	0.0	C10—C11—C18—C19	118.5 (3)
N2"—C3"—C4"—C5"	−179.7 (9)	N4—C11—C18—C23	113.8 (3)
C3"—C4"—C5"—C6"	0.0	C10—C11—C18—C23	−64.6 (4)
C4"—C5"—C6"—C7"	0.0	C23—C18—C19—C20	−1.4 (4)
C5"—C6"—C7"—C8"	0.0	C11—C18—C19—C20	175.5 (2)
C1"—N1"—C8"—C7"	−179.9 (10)	C18—C19—C20—C21	0.1 (4)
C1"—N1"—C8"—C3"	5.0 (17)	C19—C20—C21—C22	1.4 (5)
C6"—C7"—C8"—N1"	−175.1 (10)	C20—C21—C22—C23	−1.7 (5)
C6"—C7"—C8"—C3"	0.0	C21—C22—C23—C18	0.4 (5)
C4"—C3"—C8"—N1"	174.9 (11)	C19—C18—C23—C22	1.2 (4)
N2"—C3"—C8"—N1"	−5.4 (12)	C11—C18—C23—C22	−175.7 (3)
C4"—C3"—C8"—C7"	0.0	N3—N4—C24—C29	−42.7 (3)
N2"—C3"—C8"—C7"	179.7 (9)	C11—N4—C24—C29	136.9 (3)
N4—N3—C9—C10	−0.7 (3)	N3—N4—C24—C25	136.0 (3)
N4—N3—C9—C1	167.4 (5)	C11—N4—C24—C25	−44.4 (4)
N4—N3—C9—C1'	−177.1 (7)	C29—C24—C25—C26	1.3 (4)
N4—N3—C9—C1"	−172.0 (6)	N4—C24—C25—C26	−177.4 (2)
N1—C1—C9—N3	161.6 (8)	C24—C25—C26—C27	−0.4 (4)
C2—C1—C9—N3	22.5 (11)	C25—C26—C27—C28	−0.5 (5)
N1—C1—C9—C10	−34.1 (13)	C25—C26—C27—C30	176.8 (3)
C2—C1—C9—C10	−173.2 (7)	C26—C27—C28—C29	0.7 (4)
N1—C1—C9—C1'	66 (4)	C30—C27—C28—C29	−176.6 (3)
C2—C1—C9—C1'	−73 (4)	C25—C24—C29—C28	−1.1 (4)
N1—C1—C9—C1"	21.7 (12)	N4—C24—C29—C28	177.6 (2)
C2—C1—C9—C1"	−117 (2)	C27—C28—C29—C24	0.1 (4)
N1'—C1'—C9—N3	−161.6 (12)		

Symmetry codes: (i) −*x*+1, −*y*+1, −*z*+2.

### Hydrogen-bond geometry (Å, °)

**Table:**

*D*—H···*A*	*D*—H	H···*A*	*D*···*A*	*D*—H···*A*
C19—H19···O1^ii^	0.95	2.57	3.430 (3)	150

Symmetry codes: (ii) *x*−1, *y*, *z*.
